# Experimental Study of Garlic Root Cutting Based on Deep Learning Application in Food Primary Processing

**DOI:** 10.3390/foods11203268

**Published:** 2022-10-20

**Authors:** Ke Yang, Zhaoyang Yu, Fengwei Gu, Yanhua Zhang, Shenying Wang, Baoliang Peng, Zhichao Hu

**Affiliations:** 1 Nanjing Institute of Agricultural Mechanization, Ministry of Agriculture and Rural Affairs, Nanjing 210014, China; 2 Key Laboratory of Modern Agricultural Equipment, Ministry of Agriculture and Rural Affairs, Nanjing 210014, China; 3 College of Biosystems Engineering and Food Science, Zhejiang University, Hangzhou 310058, China

**Keywords:** garlic root cutting, object detection, YOLO, convolutional neural network, artificial intelligence

## Abstract

Garlic root cutting is generally performed manually; it is easy for the workers to sustain hand injuries, and the labor efficiency is low. However, the significant differences between individual garlic bulbs limit the development of an automatic root cutting system. To address this problem, a deep learning model based on transfer learning and a low-cost computer vision module was used to automatically detect garlic bulb position, adjust the root cutter, and cut garlic roots on a garlic root cutting test bed. The proposed object detection model achieved good performance and high detection accuracy, running speed, and detection reliability. The visual image of the output layer channel of the backbone network showed the high-level features extracted by the network vividly, and the differences in learning of different networks clearly. The position differences of the cutting lines predicted by different backbone networks were analyzed through data visualization. The excellent and stable performance indicated that the proposed model had learned the correct features in the data of different brightness. Finally, the root cutting system was verified experimentally. The results of three experiments with 100 garlic bulbs each indicated that the mean qualified value of the system was 96%. Therefore, the proposed deep learning system can be applied in garlic root cutting which belongs to food primary processing.

## 1. Introduction

Garlic (*Allium sativum* L.) is a traditional food grown worldwide, both as a flavoring and medicinal food [1,2,3,4,5,6,7], with a harvested area of 1.63 million hectares worldwide [8]. Currently, the harvesting and post-processing of garlic in China are primarily carried out manually, which is labor-intensive and inefficient. In the post-processing, root cutting must be carried out manually. Cutting off the garlic root reduces the probability of mildew at the bottom of the bulb due to the reduction of moisture contained in the garlic root and the soil entrained by it.

Garlic bulbs vary in shape and size, which is the main factor limiting the development of mechanized garlic root cutting. The mechanical equipment cannot accurately determine the cutting position of the root cutting knife. If a fixed root cutter is used, larger bulbs will be cut incorrectly, whereas the roots of smaller bulbs will be cut insufficiently. Furthermore, garlic bulbs are easily cankered after being cut, which affects their economic value.

In recent years, the application of computers has developed rapidly with the dramatic improvement in computing power, reduction of equipment cost, and deepening of research. It has also been increasingly used in the classification and detection of garlic. Recent studies have successfully applied deep learning to an automatic grading system for garlic, which was used to classify garlic automatically after root cutting [9]. Moreover, garlic clove direction was adjusted using machine vision [10], garlic varieties were classified using a semiconductor gas sensor [11], and the change of garlic aroma was evaluated by an electronic nose [12]. The extant research suggests that computers are increasingly used in garlic processing. However, garlic root cutting requires the computer to not only make a classification but also obtain the object location information and convert the information into the correct amount of movement of the root cutting knife. Garlic root cutting requires a complex computer system that considers accuracy, speed, and reliability. In addition, random quantities of soil are usually attached to the bulbs and garlic roots, making it challenging to perform object classification and positioning using traditional computer vision techniques [9]. Therefore, an intelligent system for garlic root cutting has not been constructed yet.

With the development of deep learning technology, the combination of deep learning and computer vision has become an effective detection method. In a previous study, deep learning was the more effective means to identify the edible safety of porcini, compared with partial least square discriminant analysis [13]. The accuracy and robustness of a model using a convolutional neural network (CNN) in the identification of hybrid okra seeds were higher than those of other models [14]. A deep neural network was used to detect weeds in a wheat field in real time [15]. Through comparison, it was found that the application of a CNN makes an important breakthrough in the detection of citrus fruit pulp [16]. Moreover, a deep learning model was more accurate than a traditional machine learning method in detecting watermelon seeds [17]. The application of a deep learning network to semantic segmentation of grape vine detection achieved higher overall performance than classical methods [18], and the proposed deep learning method achieved high accuracy in image detection of rice diseases [19].

The you only look once (YOLO) algorithm solves object detection as a regression problem, realizing the output from image data and input to object category and predicting the bounding box coordinates [20,21,22]. The mechanism of YOLO enables faster detection speed while maintaining relatively high average precision [23]. The adoption of improved YOLOv3 enabled accurate detection of tomatoes under complex environmental conditions, such as illumination changes, occlusion, and overlap [24]. The improved YOLOv4-tiny model could accurately detect table grapes in complex growth environments, with shadows of branches and leaves and overlapping fruits [25]. Online detection of defective apples was conducted based on the YOLOv4 deep learning model [26]. The YOLO + MRM model was used to detect the vertigo state of broiler chickens quickly and accurately [27], and strawberries were detected in real time based on the improved YOLOv4-tiny model [28]. Improvement of the YOLOv3 model through the MobileNetV1 network improved the detection of fish [29]. Furthermore, the YOLOv4 model was applied to detect fig fruit with small color differences from the background [30]. Another experiment showed that the precision and speed of the lightweight peanut detection model based on YOLOv3 improvement had been improved [31]. Finally, the application of the YOLOv4 model in the detection system of crab peeling achieved a high detection accuracy [32]. The above studies indicate that the YOLO model is suitable for object detection in complex environments and has excellent detection performance.

The YOLOv2 method is more accurate and faster than the YOLO method [21]. However, the YOLOv2 method uses Darknet-19 as a backbone network, and Darknet-19 suffers from low performance of feature extraction, insufficient depth of network, and easily accumulated training errors [33]; these characteristics limit its detection accuracy. The YOLOv3 method uses a deep residual network to effectively deepen the backbone network and fuses multi-scale features to effectively improve the detection capability of small and adjacent objects but performs poorly on medium and larger objects [22]. However, the network of YOLOv3 method is more complex than that of YOLOv2, and the calculation amount increases, thus reducing the detection speed [34]. A recent study showed that YOLOv2 is three times faster than YOLOv3 in non-destructive testing of fish behavior [35], and 38.47 FPS was used in benthonic organism detection (YOLOv2) (544 × 544) and YOLOv3 has FPS of 21.63 (608 × 608) [36]. On the PASCAL VOC2007 test dataset, the detection speed of YOLOv2 was nearly twice as high as that of YOLOv3 [34]. Moreover, Tiny-YOLOv2 was faster than YOLOv3 when used by a robot that detected badminton [23]. However, to the best of our knowledge, the comparison of YOLOv2, YOLOv3, and YOLOv4 in detection is limited at present. Further, the comparative research on the improvement of the YOLO series methods is limited.

Therefore, based on the comparison of YOLOv2, YOLOv3, and YOLOv4, we improved the detection performance of YOLOv2 while maintaining its high detection speed and used it for bulb detection. Based on the alignment of the upper surface of the bulb, the position of the cutting line (the lower edge line of the prediction box) is judged using the improved YOLOv2 method, and the height of the root cutter is automatically adjusted by the control system. An automatic root cutting method of garlic based on artificial intelligence, suitable for food processing, production, and combined harvest, is explored.

To the best of our knowledge, our team are the first to develop a YOLO object detection system for garlic root cutting. This study was based on our previous research. We investigated which features of the garlic plant are suitable for object detection with the purpose of root cutting and performed root cutting experiments using a classical feature extraction network [37]. We conducted an optimization study for the training parameters and investigated the detection performance of classical and lightweight network detection models for bulbs [38]. The main contributions of the work in the paper are as follows.

(1) An experimental platform consisting of equipment and a control system was built. An object detection model with a lightweight feature extraction network was preferred. The position of the cutting line was obtained by the prediction frame of the YOLO algorithm and the height of the root cutting knife was adjusted automatically. Garlic root cutting based on non-destructive detection and positioning of bulbs was achieved.

(2) The features learned by the same lightweight network before and after training were visualized and analyzed. Additionally, the features learned by different lightweight networks were visualized and analyzed. The reasons for different prediction results obtained by different object detection models were analyzed and discussed.

The remainder of this paper is organized as follows. Section 2 presents the materials and methods used in this study, Section 3 the experimental setup and results, Section 4 discussions, and Section 5 our conclusions.

## 2. Materials and Methods

### 2.1. Garlic Root Cutting Test Bed

#### 2.1.1. Introduction to Test Bed

**Figure 1:**
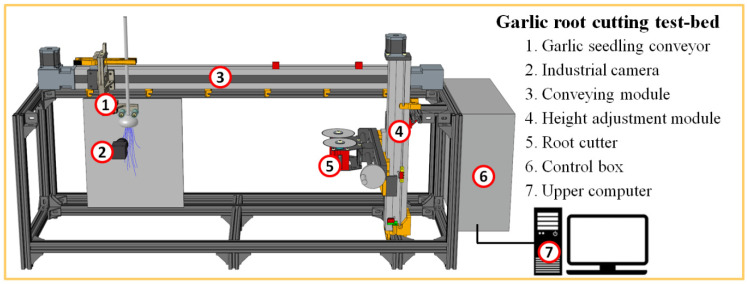
Overview of the test bed.

The garlic harvesting and root cutting device based on deep learning object measurement is constructed from a garlic seedling conveying device, root cutter, and control system. The control system for object detection and automatic adjustment of the root cutter is a set of hardware and software. The test bed is shown in Figure 1.

The deep learning-based garlic harvesting and root cutting device completes the root cutting operation through the cooperation of the garlic seedling conveying device, root cutter, and control system. After the root cutter is reset, its cutter head is in a plane with the axis line of the camera. The control system collects images at the beginning of the garlic seedling conveying and performs object detection. The height position of the root cutter is adjusted according to the cutting line predicted by the deep CNN. The garlic seedling conveying device transports the garlic seedlings forward at a set speed. In the process of conveying, the garlic root is cut by the reverse high-speed rotating root cutter, resulting in the separation of the garlic root and bulb. After the garlic seedling conveying device stops moving, it returns to the starting place, takes down the garlic seedling that has passed through the root cutting, and finally fixes the next uncut garlic seedling to enter the next working cycle. All root cutting work is completed under the control system without manual regulation. Moreover, there is no need for contact between the mechanical structure and the bulb during position detection, which avoids certain types of damage [37].

#### 2.1.2. Root Cutter

**Figure 2:**
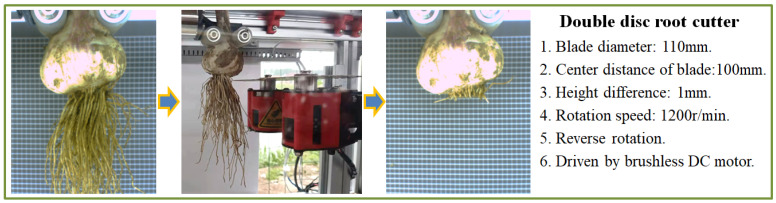
Overview of the root cutter.

To separate the garlic bulb from the garlic root effectively, a disc cutting blade with wedge edge was designed according to the characteristics of the garlic root. During work, a two-way shear force is applied to the garlic root through a pair of blades of the double disc root cutter. After being stressed, the garlic root has no swing space. The shear force acts effectively on the garlic root and cuts it off. As can be seen in Figure 2, the garlic root can be effectively cut with a double disc root cutter fitted on the test stand. The cut is straight, and the garlic root below the cut is completely separated.

#### 2.1.3. Hardware Composition of the Control System

**Figure 3:**
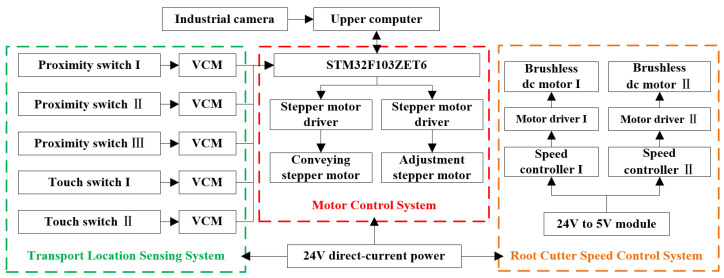
Hardware block diagram of the control system.

Figure 3 shows the hardware block diagram of the control system. The control system hardware consists of an industrial camera, transport location sensing system, motor control system, root cutter speed control system, and upper computer. The transport position sensing system senses the slider position by the proximity switches I–III arranged on the transport module and the touch switches arranged on the height adjustment module. The signal voltage of each switch is changed by the voltage conversion module to 3.3 V. The speed of the DC brushless motor is changed by changing the frequency of PWM waves emitted by the speed controller. The upper computer can complete object detection based on a deep CNN.

#### 2.1.4. Camera Calibration

**Figure 4:**
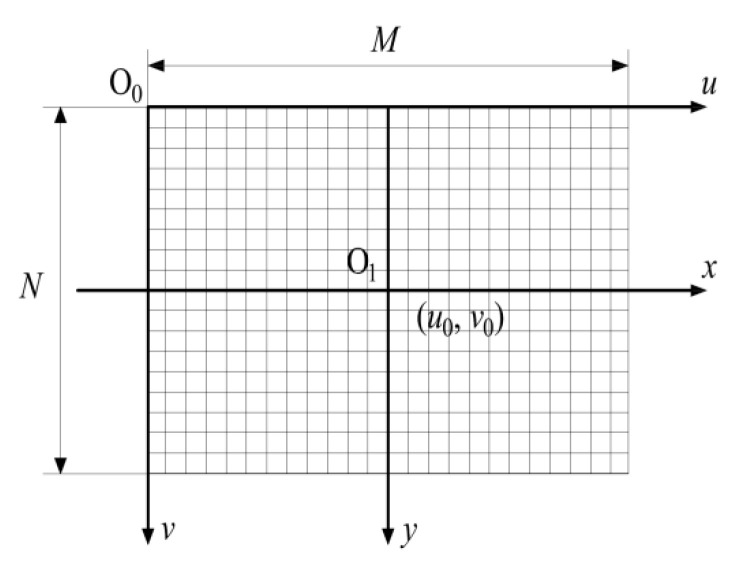
Image coordinate system.

As shown in Figure 4, *O*_0_ in the image is the origin of the pixel coordinate system, (*u*, *v*) represents the abscissa and ordinate of any pixel point. The pixel coordinates of the optical axis projection point *O*_1_ of the camera lens are (*u*_0_, *v*_0_). An image coordinate system in millimeters is established with the point *O*_1_ and the X and Y axes ((*x*, *y*) is any point in the coordinate system), and the width and height of each pixel in the image are *ρ_w_* and *ρ_h_*. The relationship is as follows [37]: $=\frac{x}{\rho_{w}}+u_{0}$;$\;v=\frac{y}{\rho_{h}}+v_{0}$. The correspondence between pixel coordinates and physical size in the height direction is$\;y=\left(v-v_{0}\right)\rho_{h}$. However, due to the distortion of the lens, it is necessary to obtain the distortion parameters of the camera and calibrate the camera to ensure the accuracy of pixel information [39].

#### 2.1.5. Microcontroller

A development board with an STM32F103ZET6 chip as a core is used as the lower computer to control the movement of the conveying and height adjusting stepping motors. STM32F10x series chips belong to ARM architecture and are developed based on the Cortex-M3 core. The maximum working frequency is 72 MHz. There are four general timers and two UARTS. They have the advantages of high performance, low power consumption, and low cost [40].

#### 2.1.6. Communication System Design

UART communication is adopted between the upper and the lower computers. STM32F103ZET6 is connected with the USB port of the upper computer through the onboard USB to RS232 converter to receive TTL level signal. The program converts the data into decimal digital command code with a value of 0–255, and the baud rate is set to 115,200 b/s.

#### 2.1.7. Design of the Control Algorithm

In the design of the control algorithm, the edge line of the boundary box predicted by the deep CNN is used as the cutting line. According to the garlic root cutting requirements, when the object of object detection is the bulb, the lower edge of the prediction boundary box is used as the cutting line.

**Figure 5:**
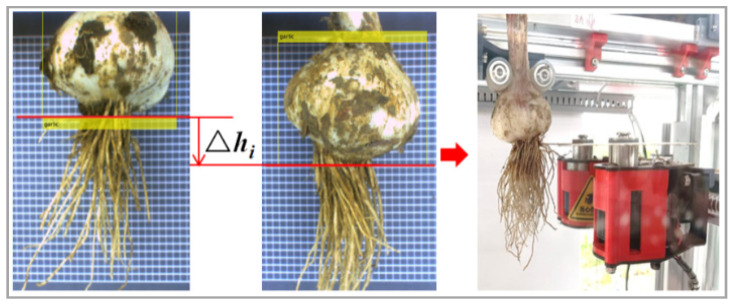
Schematic diagram of the adjustment method.

After the object detection is completed, the host computer calculates the pixel ordinate of the cutting line. Taking the bulb as the detection object, the host computer calculates the pixel ordinate of the lower edge line of the boundary box, and the position of the cutting line is shown by the red line in Figure 5.

After the previous root cutting, the upper computer converts the image coordinates of the cutting line according to the pixel coordinates of the cutting line predicted this time and feeds them back to the motor control system to adjust the height of the root cutting knife. According to the formula$\;y=\left(v-v_{0}\right)\rho_{h}$, the adjustment displacement of the height of the root cutter is $△h_{n+1}=\left(k_{n+1}-k_{n}\right)\rho_{h}\prime \;$(n ∈ N+), where ∆*h_n_*_+1_ is the coordinate difference between the *n* + 1st root cutting line and the *n*th root cutting line in the image coordinate system in the height direction. The value is below the timing cutter and upward in mm for the negative cutter. The *n*th predicted vertical coordinate of the cutting line pixel is *k_n_*, and *k_n_*_+1_ is the vertical coordinate of the cutting line pixel predicted at the *n* + 1st time; *ρh*′ is the height of each pixel in the detected image. In addition, the relationship at the first root cutting is $△h_{1}=\left(k_{1}-k_{0}\right)\rho_{h}\prime$, where *k*_0_ is set to 300 px to complete the pixel ordinates of the cut surface of the root cutter after resetting.

To obtain the value of *ρ_h_*′, it is necessary to establish the corresponding relationship between the image pixels and the actual size [41]. In this study, the image acquisition of the vertically placed scale was performed. It was found in the measurement that 159 px corresponded to 30 mm in length at the given distance; thus, the calibration coefficient of the direction of the pixels was obtained as *ρ_h_*′ = 30 mm/159 px = 0.1887 mm/px.

### 2.2. Image Acquisition

Sheyang County in Jiangsu Province is a major garlic production area in China. In this study, field tests were conducted at the test base of Nanjing Institute of Agricultural Mechanization, Ministry of Agriculture and Rural Affairs of Sheyang County, and local garlic planted in Sheyang was selected as the research sample. Garlic has a strong geographical representation and a wide planting area in Jiangsu Province. Field tests were conducted in May 2021 with garlic from a field located at the coordinates of 33°51′56″ N, 120°13′49″ E. The parameters of the industrial camera used to acquire the images are shown in Table 1.

#### 2.2.1. Image Data

To provide reliable location information to the computer, the images contained only one garlic per image. The collected dataset contained 2500 non-repetitive garlic images; their collection was conducted over 3 days from 8 a.m. to 7 p.m. This enabled the dataset to contain different levels of brightness and ensured the adaptability of the object detection method on the test bed to different illuminations. Owing to the absence of any supplementary lighting measures, the levels of object brightness in the dataset differ considerably. 

**Figure 6:**
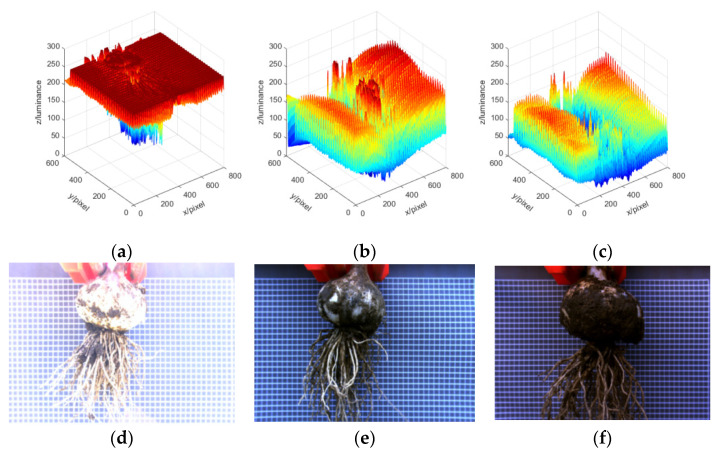
Diagram of brightness level differences: (**a**) corresponds to (**d**), (**b**) corresponds to (**e**), (**c**) corresponds to (**f**); (**d**) the average value of Y is 224.4; (**e**) the average value of Y is 100.5; and (**f**) the average value of Y is 58.7.

To facilitate visualization and quantitative analysis of the differences of brightness levels, the acquired image was converted from the RGB color space to the YUV color space [42], where Y represents the brightness information of the image and the differences of Y values of different images are shown in Figure 6. It can be seen that the brightness of the bulb and garlic root in Figure 6d was the highest, and the corresponding brightness grid curve is shown in Figure 6a. The local brightness of the bulb and garlic root in Figure 6b corresponding to Figure 6e was higher than the background brightness, and the brightness of the bulb and garlic root in Figure 6c corresponding to Figure 6f was lower. The large differences in brightness levels increase the difficulty of object detection.

#### 2.2.2. Partition of Datasets

From the dataset of 2500 images, 500 images were selected randomly as test data, and the remaining 2000 images were used as training data for the object detection algorithm. The ratio between the training set (1400 images) and the verification set (600 images) in the training data was 7:3. 

### 2.3. Pretreatment

#### 2.3.1. Image Annotation

**Figure 7:**
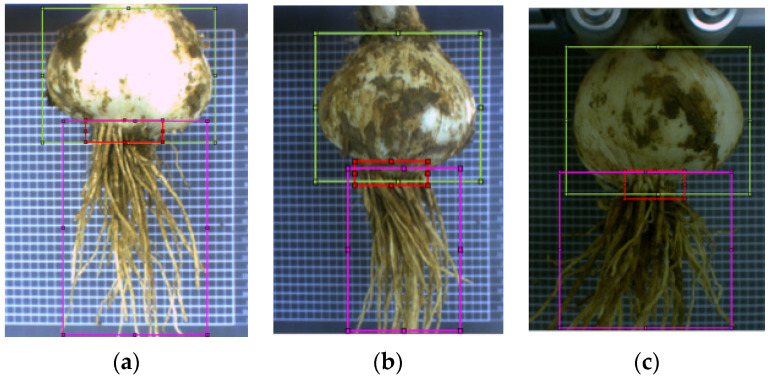
Comparison chart of different levels of object brightness: (**a**) high, (**b**) medium, and (**c**) low.

A YOLO model detects the object in the test data according to the label box information of the training data and generates a prediction box. Considering the input size, training time, and computer performance of the network model, the image resolution of the training data was adjusted to 224 × 168 px before image labeling. Then, the training data were labeled, and the labeled file was saved in an XML format. Based on the collected images, the optional detection objects were bulb, root plate, and garlic root. In Figure 7, the bulb is marked with a green box, root disc with a red box, and garlic root with a purplish red box. It can be seen that when the bulb was used as the detection object, the label box contained the entire bulb, and the upper edge lines of the label boxes of the root disc and garlic root passed through the bulb (Figure 7). Furthermore, it was found that when the root disc was used as the object, multiple prediction boxes were prone to errors. 

Therefore, the bulbs were selected as the only detection object, and only the bulbs were labeled in the training data. The labeling ensured that each bulb was framed in a label box and occupied as much area as possible in the label box. In the classification stage, the area detected by the YOLO algorithm model was considered a bulb, and the image was divided into “bulb” and “non-bulb” areas to evaluate the effectiveness of this algorithm.

#### 2.3.2. Data Augmentation

Overfitting may occur during the training of a deep CNN. An effective way to avoid overfitting is to increase the amount of training data [43,44,45]. However, the acquisition of data requires considerable human and financial resources, and in some cases, images cannot be obtained. Therefore, data enhancements are generally used to expand datasets. In this study, mirroring, hue, saturation, and exposure changes were used for data enhancement [46,47]. We performed mirroring, hue, saturation, and exposure changes once each for each training set image (1400 images in total) in the experiment. Thus, the new training set contains 5600 data-enhanced images.

### 2.4. Lightweight CNNs

Although a CNN can be used in many image recognition tasks, its large model files and high computational load lead to slow object detection [48]. As the application of CNN has become increasingly popular, the need to improve the speed of object detection has become increasingly urgent. The garlic root cutting device requires high precision and efficiency and especially high detection speed. To address the low speed problem, researchers have designed lightweight networks with fast convolution and compression schemes. In this study, three representative lightweight networks (SqueezeNet [49], ShuffleNet [50], and MobileNetV2 [51]) were selected for training and research.

### 2.5. Prediction of the Bounding Box Location

**Figure 8:**
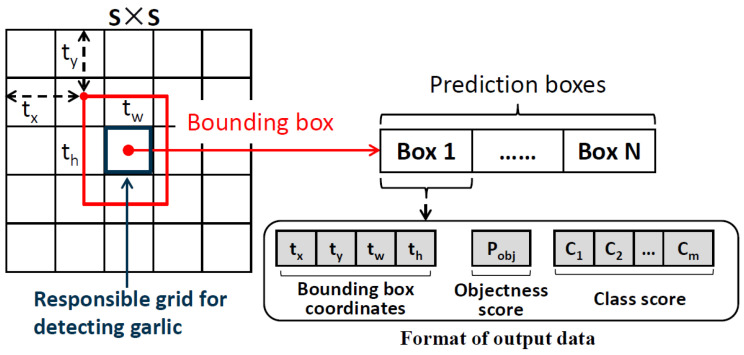
Model prediction layer.

The YOLO network model is a one-stage method. In the YOLOv2 model, the backbone network divides the input image into S × S grids, and the feature map size is equal to the grid size. The YOLOv2 model provides S × S × ((4 + 1 + m) × N) tensor output with N bounding boxes per cell and 4 + 1 + m predictions per bounding box. The predicted values for each bounding box include the pixel coordinates (*t_x_*, *t_y_*), width (*t_w_*) and height (*t_h_*), objectness score (*P_obj_*), and class score (*C*_1_, *C*_2_, …, *C_m_*) of the upper left corner of the bounding box. Ultimately, only the best bounding box is retained using the non-maximum suppression method. The objectness score indicates whether the object exists in the bounding box (with a value of 0 or 1), and class score is the confidence level that the object belongs to a category. Therefore, YOLOv2 detects the bulb, and the pixel ordinate of the cutting line is *t_g_ = t_y_ + t_h_*, which is a determined value [20,21] (Figure 8).

The total loss in the training process of the detection model is represented by *L_all_*, which is composed of boundary box detection loss (*L_IOU_*), confidence detection loss (*L_confidence_*), and classification loss (*L_class_*) [20,21]: *L_all_* = *L_IOU_* + *L_confidence_* + *L_class_*.

## 3. Experiment

### 3.1. Model Training and Testing

In this study, three classic networks, ResNet50 [33], GoogLeNet [52], and AlexNet [53], were also compared. The experiment included the comparison of YOLOv2, YOLOv3, and YOLOv4 algorithms. By comparing lightweight networks with classical networks, the performance and advantages of different network types were analyzed. Meanwhile, the models with different algorithms were compared to lay a foundation for selecting a suitable model for garlic object detection tasks.

**Figure 9:**
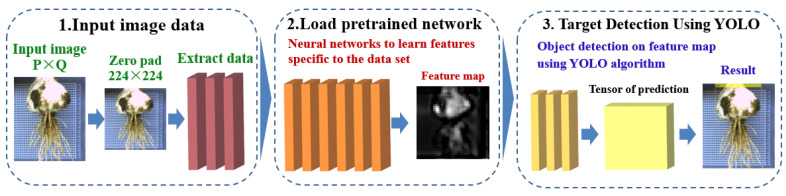
Pretrained network training.

The process of using the migration learning training object detection model is shown in Figure 9. The random gradient descent training network with momentum was used with a momentum of 0.9 and learning rate of 1 × 10^−3^ to perform the object classification of one object (bulb only); see Table 2 for specific values. The GPU used was NVIDIA GeForce GTX1650 (4GB GDDR6 memory).

### 3.2. Training Results and Evaluation

In this study, garlic plants were pulled out without any treatment, and a certain random amount of soil that adhered to the surface of the bulb or between the roots of garlic was left on the plant, which made the detection more difficult. The accuracy of object detection was measured by average precision (AP) when IoU = 0.5. Furthermore, the storage space and training time of the model were also examined. Comprehensive evaluation of the training process was conducted to select a suitable detection model. The value of AP is equal to the area under the precision–recall curve.

When evaluating the detection model, we examined the confidence score (class score) and detection time required for object detection. In addition, as garlic root cutting based on object detection is the focus of this study, the reliability of the predicted cutting line was evaluated, and the position of the cutting line was considered the decisive factor affecting the quality of root cutting. Therefore, we studied the cutting line positions of different models and backbone networks to determine which backbone network had more reliable cutting lines. When investigating the differences of boundary box positions, the average and standard deviation of the predicted cutting line positions were examined to evaluate the performance of the detection model.

#### 3.2.1. Comparison of Results of Different Model Tests

To evaluate the performance of the models, YOLOv2, YOLOv3, and YOLOv4 models based on different backbone networks were compared. To ensure the consistency of training parameters of various YOLO algorithms, the total loss of each model was stable below 0.5 at the later stage of training as the basis for training. Table 2 shows the structural comparison of different models, which shows that the average accuracy of YOLOv4-tiny-COCO was 99.97% and that of the YOLOv2-MobileNetV2 model was 99.15%. Among the 12 models, YOLOv3 and YOLOv4 models had multiple detection heads, and the number of extracted feature maps was equal to the number of detection heads. YOLOv3 and YOLOv4 algorithms implemented multi-scale feature fusion. The model was trained and 12 bulb detectors were obtained.

**Figure 10:**
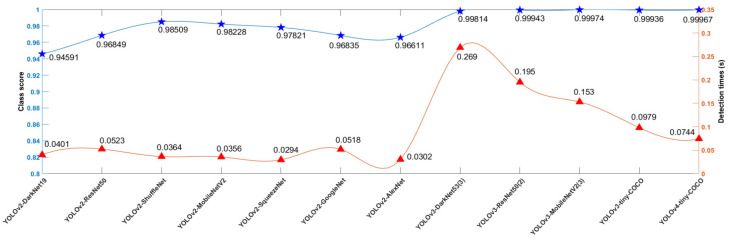
Comparison of model test results.

The confidence scores and detection times of the 12 detectors on the test set were determined (Figure 10). For suitable reaction speed, the garlic root cutting system requires the detector to have a high detection confidence score, and the detection time should be the shortest possible. Among the 12 compared models, the detection time of YOLOv2-SqueezeNet was the shortest, and YOLOv2-MobileNetV2 was only 0.0062 s slower than YOLOv2-SqueezeNet, which was almost negligible. However, the confidence score of YOLOv2-MobileNetV2 was 0.00407 higher than that of YOLOv2-SqueezeNet and higher than that of YOLOv2-ShuffleNet, which indicates that the YOLOv2-MobileNetV2 model has the most suitable combination of detection confidence score and detection speed.

**Figure 11:**
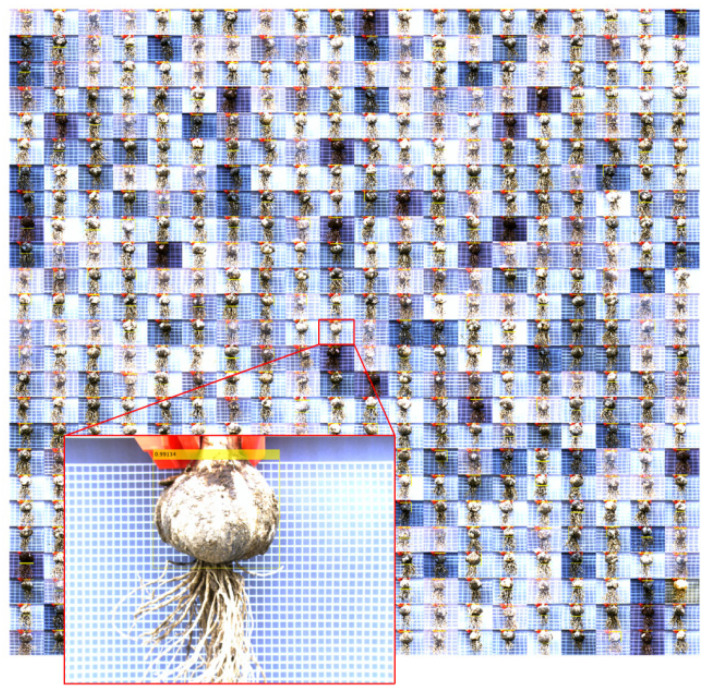
Test results of YOLOv2-MobileNetV2.

Therefore, the YOLOv2-MobileNetV2 model achieved the most suitable performance from the 12 models and can realize fast and accurate detection of bulbs. The test set consisted of 500 images with different brightness, bulb shapes, and amounts of soil on the bulb surface. The results of the YOLOv2-MobileNetV2 detection on the test set are shown in Figure 11. In each image, the bulb object location was accurately detected, and a confidence score was output, indicating that the bulb detector trained by the YOLOv2-MobileNetV2 model had good generalization, and the model had not been fitted.

**Figure 12:**
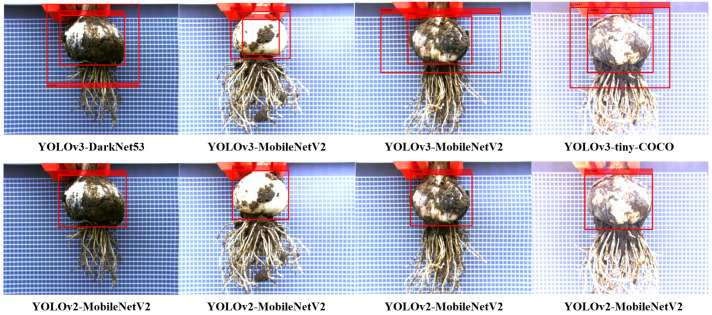
Comparison of test results for improved models.

After statistical analysis, the average width and height of the prediction box obtained by YOLOv2-MobileNetV2 for bulb object detection in test data (500 images) were 307 px and 245.6 px, respectively. From the pixel size, the bulb in the image is a single large object [54]. During the test, it was found that the YOLOv3 algorithm provided several prediction boxes incorrectly when detecting bulbs. As shown in Figure 12, although there was only one bulb in the image, YOLOv3-DarkNet53, YOLOv3-MobileNetV2, and YOLOv3-tiny-COCO all output incorrect results with two prediction boxes. However, YOLOv2-MobileNetV2 correctly detected the images where the YOLOv3 algorithm made errors and did not output multiple prediction boxes in the test data (500 images). Tests showed that YOLOv2-MobileNetV2 was less prone to errors than YOLOv3-based models in bulb detection.

#### 3.2.2. Visualization of Feature Maps

To compare the differences between different CNNs and their differences before and after migration learning training, the features extracted by the CNNs were visualized. Generally, visualization of features extracted by CNNs is considered an appropriate method for evaluating model performance [55].

Different CNNs learn different characteristics of images. The learning characteristics of a CNN will change before and after training. As the training proceeds, the CNN learns the characteristics of images by itself, but sometimes, it is not clear what it learned during the training process. In the YOLO algorithm, each layer of the CNN outputs an active 3-D object, which is sliced along the third dimension of the active 3-D object (i.e., the channel), and each slice corresponds to a single filter generated by that layer. Further, the active 3-D volume output from the deeper neural network is an advanced combination of the shallower learning characteristics.

**Figure 13:**
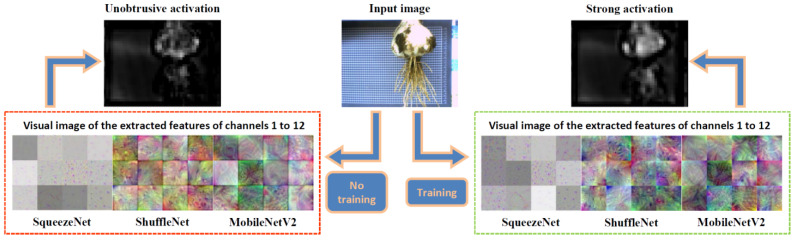
Comparison of model test results before and after training.

We compared visualized images of the features extracted from the output layers of different backbone networks on channels 1 to 12, as shown in Figure 13. The CNN before training had the ability of classification owing to transfer learning. There are obvious differences in image features extracted by YOLOv2-SqueezeNet, YOLOv2-ShuffleNet, and YOLOv2-MobileNetV2 in the output layer before and after training. Moreover, there are significant differences among the filters of the three backbone networks. Differences in features extracted from different backbone networks cause deviations in the predicted cutting line position. In addition, Figure 13 shows a visual difference in the activation of the backbone network to the object area before and after training. It can be seen that the bulbs are strongly activated after the backbone network has been trained. Better trained activation of garlic features in images is conducive to improving the accuracy of object detection.

#### 3.2.3. Difference in Cutting Line Position

**Figure 14:**
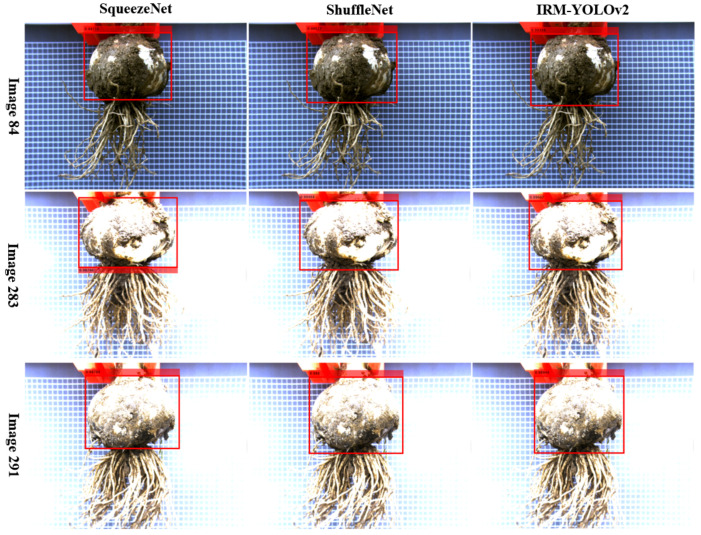
Prediction of cutting line location by different backbone networks.

As the YOLOv2 model had a suitable combination of detection speed and confidence score to meet the requirements of garlic root cutting, only the YOLOv2-based models are discussed in this section. As shown in Figure 14, the YOLOv2 models of the three backbone networks, SqueezeNet, ShuffleNet, and MobileNetV2, differed greatly in the cutting line positions predicted in Image84, Image283, and Image291. It can be seen that different backbone networks predicted different cutting line positions. As, in some instances, the cutting line passed through the bulb, the position of the cutting line must be studied.

From the point of view of object detection, the position of the boundary box predicted by the YOLOv2 model is directly related to the position of the annotation box in the training data. We guarantee that the border of the label box did not pass through the bulb when labeling the training data. Therefore, the occurrence of the cutting line passing through the garlic bulb in the object detection result was caused by the prediction of the YOLOv2 models.

**Figure 15:**
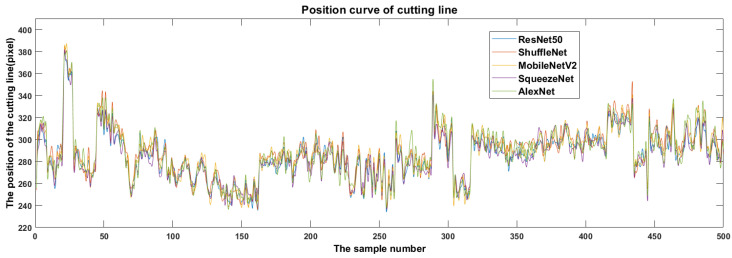
Cutting line location differences predicted by different backbone networks.

To find a reliable detection model and avoid cutting the bulbs during root cutting, it is necessary to study the differences in cutting line positions. First, the cutting line positions predicted by various YOLOv2 models on the test set were analyzed statistically. In Figure 15, the horizontal coordinate represents the serial number of the sample and the vertical coordinate the predicted vertical coordinate value of the cutting line pixel. Thus, when object detection is performed on the same image, the larger the ordinate value, the greater the distance between the cutting line and the bulb, which is beneficial to avoid scaling the bulb. The trend of the curve change of the cutting line position of each YOLOv2 model is basically the same, but there are obvious fluctuations among the curves (Figure 15). This indicates that when object detection was performed on the same image, the predicted cutting line positions might have been significantly different.

Then, the prediction bias formula for the vertical coordinate values of the cutting line pixels at each sample point is $\sigma_{jk}=x_{jk}-{\bar{x}}_{j}$, ${\bar{x}}_{j}=\frac{1}{m}\sum_{i=1}^{m}x_{ji}$, where *j* is the number of sample points (*j* = 1, 2, …, 500), *m* is the type of the model (*m* = 5), *k* is the number of the model (*k* = 1, 2, …, 5), $x_{jk}$ is the pixel coordinate value of the cutting line predicted by the *k*th model at the *j*th sample point (image), ${\bar{x}}_{j}$ is the average of the cutting line pixel coordinates predicted by each model for the *j*th sample point (image), and $\sigma_{jk}$ is the deviation of the pixel coordinate values of the cutting line predicted by the *k*th model at the *j*th sample point (image).

**Figure 16:**
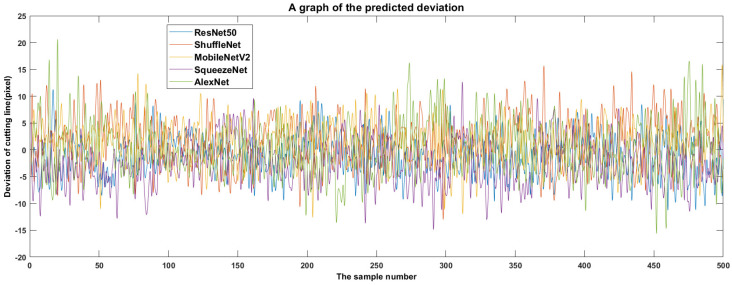
Cutting line position deviation predicted by different backbone networks.

The distribution of prediction bias for each YOLOv2 model can be seen in Figure 16. Assuming that the predicted cutting line pixel coordinate average ${\bar{x}}_{j}$ is considered to be the exact cutting line location, a larger $\sigma_{jk}$ indicates that the predicted cutting line is farther from the bulb, and a smaller $\sigma_{jk}$ indicates that the predicted cutting line is closer to the bulb.

**Figure 17:**
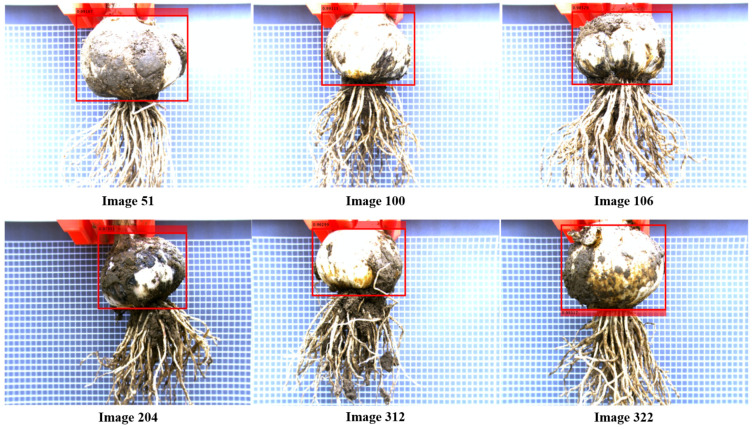
Forecast results of YOLOv2-MobileNetV2.

The farther the cutting line is from the bulb, the lower the risk of bulb cuts, and the more reliable the cutting line. The closer the cutting line is to the bulb, the more likely will the bulb be cut, and the more unreliable the cutting line. As shown in Figure 16, the prediction bias of YOLOv2-MobileNetV2 at sample points 51, 100, 106, 204, 312, and 322 is significantly smaller than that of other models. Although YOLOv2-MobileNetV2 predicted cutting lines close to the bulb at sample points 51, 100, 106, 204, 312, and 322, none of the cutting lines penetrated the bulb (Figure 17).

**Figure 18:**
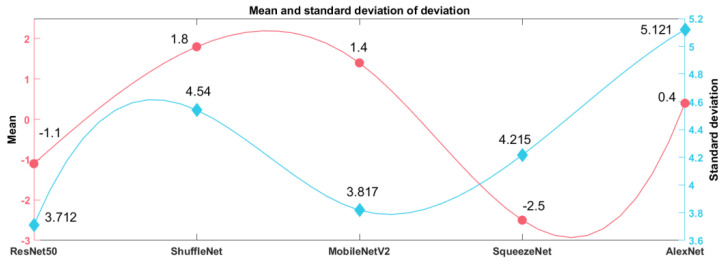
Statistical analysis of prediction results.

Finally, the deviations of the different models in Figure 16 were complex, so a statistical analysis of the deviations was conducted to determine which model to choose. Figure 18 was obtained by statistical analysis of the deviations of the vertical coordinate values of cutting line pixels predicted by the five YOLOv2 models. The order of the average values of prediction deviations was ShuffleNet, MobileNetV2, AlexNet, ResNet50, and SqueezeNet from largest to smallest (Figure 18). The standard deviations of prediction deviations were in the order of ResNet50, MobileNetV2, SqueezeNet, ShuffleNet, AlexNet from the smallest to largest. The larger the mean of the prediction bias, the farther the cutting line is from the bulb, and the better the reliability. However, as can be seen from Figure 16, there are prediction errors in each model. Considering the stability and reliability of predictions, the smaller the standard deviation of prediction errors, the more reliable the prediction. Considering both the mean and standard deviation of prediction bias, YOLOv2-MobileNetV2 has the best reliability from the five YOLOv2 models in predicting cutting lines.

#### 3.2.4. Root Cutting Test

After the comparison, YOLOv2-MobileNetV2 was chosen as the object detection model for the root cutting test considering the accuracy, size, confidence score, detection speed, and reliability of the predicted cutting line.

**Figure 19:**
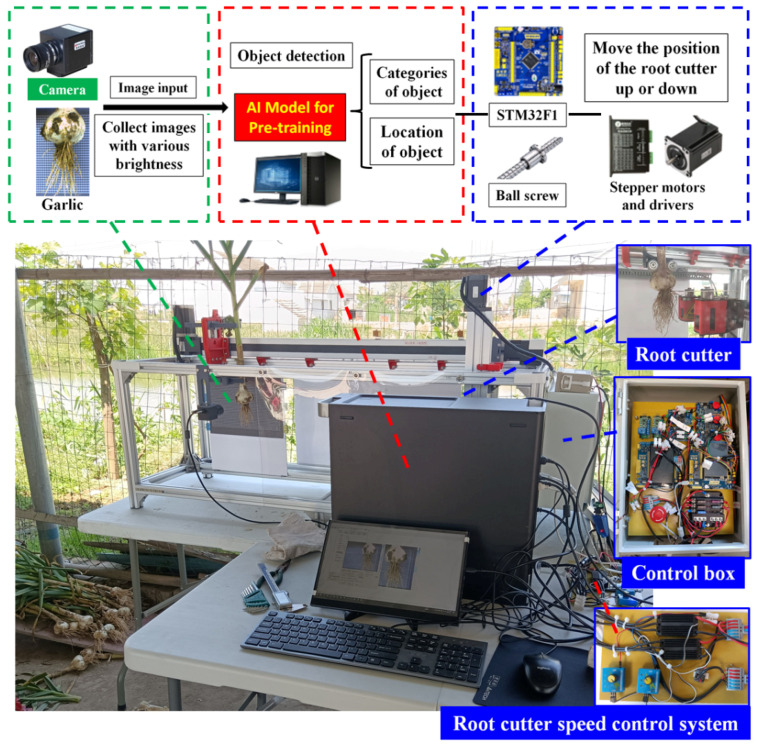
Field test.

During the test, the conveying speed was 0.7 m/s, and the rotating speed of the cutter’s disc was 1200 r/min. There were three groups of experiments, each with 100 samples. The brightness of the collected images was determined by weather conditions. In this study, samples with cut bulbs and remaining garlic roots no longer than 10 mm in length were considered qualified, as the bottom of the bulbs after root cutting would not be mildewed. The qualified rate of root cutting was obtained through observation and measurement. The formula for calculating the qualified rate of root cutting was $\alpha =\frac{N_{1}}{N_{0}}\times 100\%$, where N_1_ is the number of qualified samples of root cutting, and N_0_ is the total number of samples. The field test system is shown in Figure 19.

**Figure 20:**
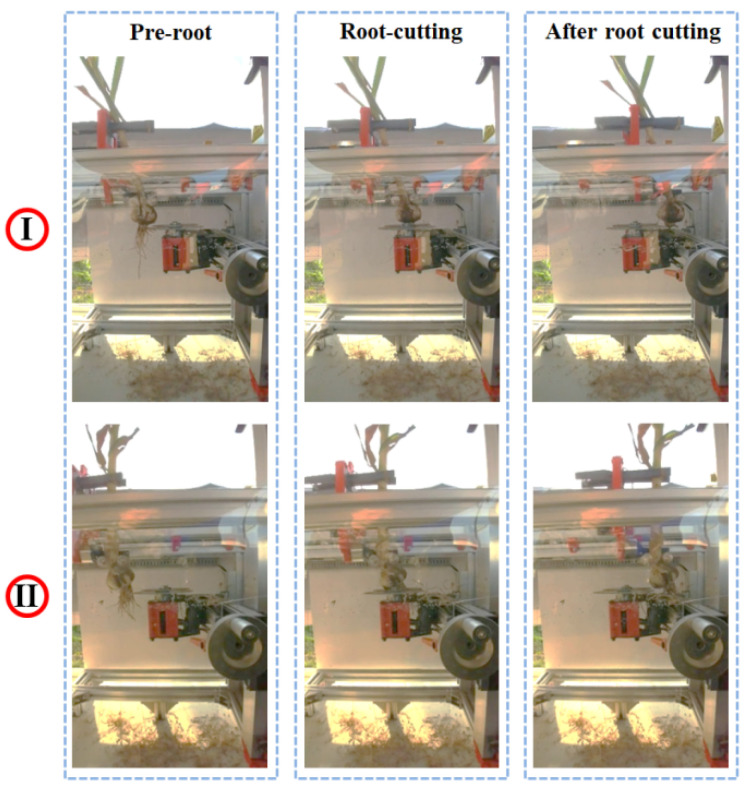
Comparison of before and after root cutting: (I) before, during, and after a root cutting process; (II) before, during, and after a root cutting process.

The comparison of the bulbs before and after the test root cutting is shown in Figure 20. It can be seen that the garlic root has been effectively cut by the root cutter. Table 3 shows the results of the experiments; the mean confidence score of detecting a bulb object was 0.98099, the mean qualified rate of root cutting was 96%, and the mean detection time was 0.0354 s. In the root cutting test, two types of unqualified samples were observed: cut bulbs and excessively long residual roots. Unqualified samples reduced the qualified rate of root cutting. In a follow-up study, feasible measures to further improve the qualified rate of root cutting will be found.

## 4. Discussion

The error in this work on garlic root cutting based on deep learning may come from several sources. We mainly analyzed the detection errors that exist in different target detection models when performing target detection. In addition, the errors include the motion error of the actuator, the rounding error of the position of the prediction frame in the pixel coordinate system, etc. The rounding error of the position of the prediction frame in the pixel coordinate system is no more than 1 pixel and can be ignored for small values. Here, we mainly discuss the motion error of the height adjustment module. The height adjustment module mainly contains a stepper motor (57CM18, Rtelligent, Shen Zhen, China) and an enclosed single-line rail slide (DXG40-1610-400, Hengchuangchuandong, Tai Zhou, China). The stepper motor (57CM18, Rtelligent, Shen Zhen, China) has a step accuracy of ±5% (full step, no load), 2000 pulse/rev. The enclosed single-line rail slide (DXG40-1610-400, Hengchuangchuandong, Tai Zhou, China) has a screw. The accuracy is 0.03 mm~0.05 mm. It can be seen that the combination of a stepper motor and an enclosed single-line rail slide table can achieve high-accuracy motion control at a low cost. In addition, the contact switch (SS-5GL2, OMRON, Japan) used for resetting the root cutter has a positioning error of 0.4 mm, ensuring reliable resetting.

Since garlic cutting requires the smallest possible motion error of the root cutter, subsequent tests will focus on the reliability of the combination of the stepper motor and the enclosed single-line rail slide. If the root cutter requires higher motion accuracy, a servo motor drive will be considered.

## 5. Conclusions

In this study, deep learning was innovatively introduced into the automatic root cutting of garlic for the first time. A transfer learning-based YOLOv2-MobileNetV2 method was proposed for non-destructive bulb object detection. The average accuracy of the YOLOv2-MobileNetV2 model was 99.15%, the detection time was 0.0356 s, and detection was reliable. The brightness of training and test data was quantitatively analyzed by color space conversion. The feature visualization of the output channel of the backbone network showed the advanced features of network learning before and after training as well as the advanced features of different methods of network training. The position differences of cutting lines predicted by different backbone networks were visually analyzed. Its excellent and stable performance indicated that YOLOv2-MobileNetV2 had learned the correct features in data with different brightness levels. Based on this, YOLOv2-MobileNetV2 was successfully applied to the garlic root cutting test stand to complete the automatic adjustment of the root cutting knife, and the effect of the automatic adjustment was verified. The mean confidence score of detecting a bulb object was 0.98099, the mean qualified rate of root cutting was 96%, and the mean detection time was 0.0354 s. In conclusion, all the results show that the proposed garlic root cutting method based on deep learning is effective and suitable for automatic garlic root cutting. Deep learning has a wide range of application scenarios in food processing.

## Figures and Tables

**Table 1:** Camera parameter setting.

Variable	Value/State	
Camera model	Minsvision MS-UB500C	
Image size	800 × 600 px	
Aperture D/f′	F1:1.6	
Focal length (mm)	6–12	
Format	1/2″	
Horizontal field	53°–28°	
Operation mode	Zoom	Manual
	Focus	Manual
	Iris	Manual
Macro	Off	
Image type	bmp	

**Table 2:** Comparison of model structures.

Algorithm	Backbone Network	AP (%)	Test Heads	Network Input	Size of Feature Map	Size (MB)	MaxEpochs	MiniBatchSize	Anchorboxes	Training Time/(s)
	DarkNet19	94.93	1	224 × 224 × 3	7 × 7	137	60	16	7	4970
	ResNet50	97.75	1	224 × 224 × 3	14 × 14	97.6	60	16	7	893
	ShuffleNet	98.67	1	224 × 224 × 3	14 × 14	6.1	60	16	7	3665
YOLOv2	MobileNetV2	99.15	1	224 × 224 × 3	14 × 14	23.5	60	16	7	4921
	SqueezeNet	97.98	1	224 × 224 × 3	14 × 14	18.7	60	16	7	2901
	GoogLeNet	96.93	1	224 × 224 × 3	14 × 14	56.7	60	16	7	4101
	AlexNet	96.79	1	227 × 227 × 3	13 × 13	12.5	60	16	7	2647
	DarkNet53	98.94	3	256 × 256 × 3	32 × 32/16 × 16/8 × 8	236	25	8	9	13,987
YOLOv3	ResNet50	99.79	2	224 × 224 × 3	28 × 28/14 × 14	111	25	8	6	11,281
	MobileNetV2	99.82	3	224 × 224 × 3	28 × 28/14 × 14/7 × 7	42	25	8	9	9098
YOLOv3-tiny-COCO		99.91	2	224 × 224 × 3	14 × 14/7 × 7	30.8	25	8	6	3108
YOLOv4-tiny-COCO		99.97	2	416 × 416 × 3	26 × 26/13 × 13	20.9	30	8	6	6759

**Table 3:** Results of the root cutting test.

	Experiment			Mean Value
	1	2	3	
Confidence score	0.97814	0.97894	0.98589	0.98099
Qualified rate (α)/%	95	97	96	96
Detection time/s	0.0354	0.0352	0.0356	0.0354

## Data Availability

The data collected in this research are available when required.
